# The Blind Men and the Piwi Elephant—Opening a Can of Worms

**DOI:** 10.1371/journal.pbio.1002062

**Published:** 2015-02-10

**Authors:** Roland G. Roberts

**Affiliations:** Public Library of Science, Cambridge, United Kingdom

Biologists’ pragmatic need to use model organisms means that they often have to treat the tree of life as the proverbial blind men treated the elephant. The blind men struggled to reconcile their seemingly irreconcilable descriptions of tusks, tail, trunk, and leg, but biologists frequently study a phenomenon in organisms from distant branches of the tree (for example, humans, mice, flies, and worms) and then confidently infer its universality. The parsimony with which evolution operates means that most of the time they’ll be correct; sometimes, however, it may be beneficial for them not to simply compare notes and then jump to conclusions but rather to gradually feel their way along the tree branches and see whether they ever encounter each other.

This synopsis recounts a cautionary and intriguing tale of just how deceptive long-distance similarities between such disparate animal models can be. It involves the constant firefight that living things wage against the relentless assault on their genomes by transposons. These “selfish genes,” akin to computer viruses, lurk in our cellular software until their copy/paste instructions are activated by transcription into RNA, resulting in potentially damaging insertions into other parts of the genome. This is clearly undesirable, and—unsurprisingly—organisms have evolved ingenious ways to suppress transposon activity, especially in the reproductive cells, in which a transposition event would affect not only the organism in question but also subsequent generations.

A key way of policing the activity of transposons involves a protein called Piwi, which belongs to a large tribe of RNA-binding proteins called the Argonaute family. The Argonaute proteins have a slot that accommodates a small RNA molecule (20–30 nucleotides long) with its sequence of bases sticking outwards so that they can recognize longer complementary RNA molecules by base pairing. This pairing targets the Argonaute proteins to the complementary RNAs, allowing the Argonautes to cleave or sequester the RNAs or to silence their genes. Different Argonautes bind subtly different small RNAs; those favored by Piwi are called Piwi-interacting RNAs (piRNAs), and they direct Piwi to silence transposons.

The Piwi-piRNA system is seen in humans, mice, flies, and worms, and (see above!) is therefore inferred to be near universal among animals. There are some differences in the way that they are made and in the exact downstream consequences of their activity, but essentially the piRNAs are always made from snippets of transposon complementary RNAs, they bind Piwi, and they ultimately silence the transposon from which they were derived. They even share details like a uracil base at the front end and a methyl group at the back end.

But what if the biologist who examines the worm (or, more specifically, *the* worm, *Caenorhabditis elegans*), instead of conferring with the biologist who studies the fly, tries to gradually feel his way along the tree of life towards her? This is essentially what Peter Sarkies, Eric Miska, and colleagues have done in a new study just published in *PLOS Biology*. They start from the well-known situation in *C*. *elegans* and then do a broad study of small RNAs—and the proteins that make and then use them—across the entire nematode phylum. This survey of the workhorse worm’s phylogenetic neighborhood reveals an unsuspected degree of evolutionary dynamism, not only in the Piwi-piRNA system, but also in other small RNA pathways and other transposon management schemes.

The nematodes are considered to fall into five groups, or clades, named Clades I to V (*C*. *elegans* itself is in Clade V), so the authors took 11 species of nematode that cover these clades representatively and sequenced their repertoire of small RNAs. They also used already-available data on two further species. As expected, they found numerous piRNAs, or “21U-RNAs” (so named because they’re 21 nucleotides long and start with a uracil), in *C*. *elegans* and in its fellow Clade V members. However, worms from the other clades had neither 21U-RNAs nor the slightly longer piRNAs found in some non-nematode animals.

Gratifyingly, the worm version of the Piwi protein (PRG-1) was also missing outside Clade V, as was HENN-1, the enzyme that sticks the characteristic methyl group onto piRNAs. Thus, this pathway—the piRNAs themselves, an enzyme that helps make them, and the protein that uses them to silence transposons—is present in non-nematode animals, including fellow ecdysozoans like insects and tardigrades, but is only found in one specific group of nematodes. The rather unparsimonious explanation for this pattern—and one for which the authors provide compelling evidence—is that the Piwi/piRNA pathway must have been present in the great-granddaddy of all nematodes but independently lost in multiple nematode lineages during evolution.

Does this mean that the genomes of worms from Clades I, II, III, and IV are riddled with rampant transposons, unconstrained by piRNAs? Apparently not. It was already known that piRNAs in *C*. *elegans* silence transposons indirectly via another family of small RNAs called “22G-RNAs” (yes, they’re 22 nucleotides long and start with a guanine). The authors checked to see whether 22G-RNAs were also present in the non-Clade V worms, exploiting the fact that these molecules, unlike most other small RNAs, have triphosphate groups at the front end.

**Fig 1:**
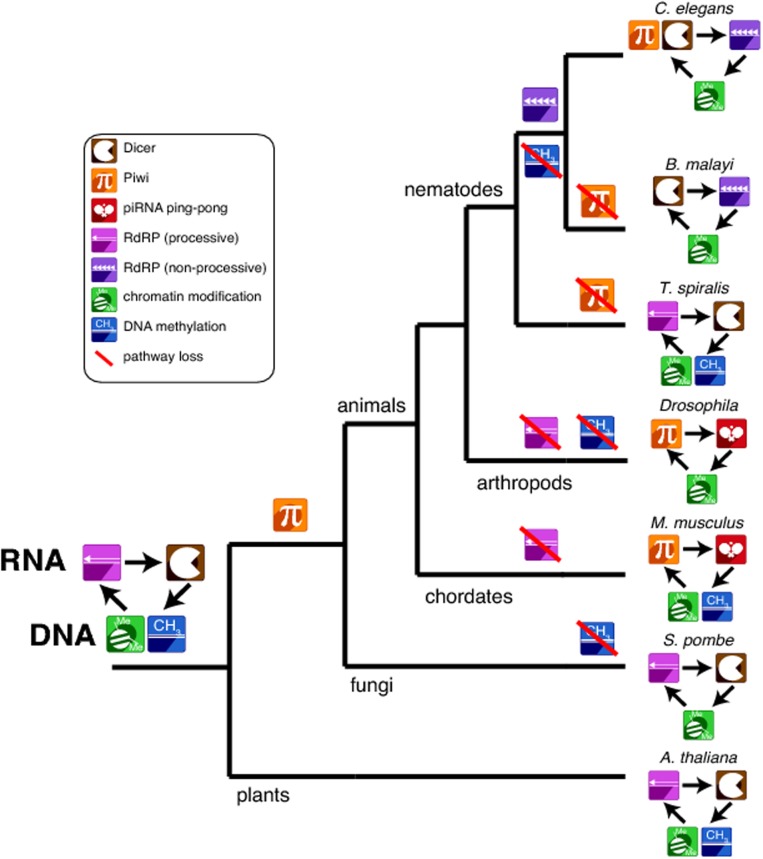
Dynamic evolution of transposon-silencing pathways. The activity of transposons is recognized at the level of RNA, and transposons are silenced either posttranscriptionally (through RNA cleavage) or transcriptionally (through chromatin modification and/or DNA methylation). Symbols indicate pathways used for defense against transposons in each species; note the differences within the nematode phylum—Clade I and II nematodes are represented by *T*. *spiralis*, Clades III and IV by *B*. *malayi*, and Clade V by *C*. *elegans*.

Yes, Clade III and Clade IV worms, like the Clade V lab workhorse *C*. *elegans*, have 22G-RNAs. This seems to suggest that while the silencing of transposons by 22G-RNAs in Clade V worms needs some upstream help from piRNAs, the Clade III and IV 22G-RNAs can get by on their own (this group of animals is represented by the Clade III nematode *Brugia malayi* in Fig. 1). Indeed, the 22G-RNAs in these piRNA-less worms do seem to consist of transposon-derived sequences and so are likely to be performing a similar function.

But the 22G-RNA system is missing from Clades I and II, implying that it arose anew in a common ancestor of Clades III–V. The authors duly show that that the three RNA-directed RNA polymerases (RdRPs) needed to make 22G-RNAs from transposon RNA are also only present in these three clades—these enzymes seem to have a specially inserted sequence that the authors speculate may adapt them to the manufacture of 22G-RNAs by liberating them from the need for a primer.

This still leaves Clades I and II (represented in Fig. 1 by the Clade I nematode *Trichinella spiralis*) without an obvious transposon-silencing mechanism. The authors find that although there are no obvious 21U-RNAs (piRNAs) or 22G-RNAs, these worms contain a host of transposon-derived small RNAs that have properties distinct from either of those categories. Instead, these are 23–25 nucleotides in length, are unmethylated, and appear to have originated as sense-antisense pairs with staggered ends—a characteristic of small RNAs that have been snipped out of a longer double-stranded RNA by an enzyme known as Dicer (maker of the most famous class of small RNAs—the microRNAs).

The authors in fact build an intriguing circumstantial case that in Clade I and II nematodes, RdRPs copy the single-stranded transposon RNA into double-stranded RNA; this is then snipped into small RNAs by Dicer and goes on to direct RNA-directed methylation of the original transposon DNA—a form of epigenetic silencing. Although unproven, this is tantalizing, because similar systems are found in plants and fungi (compare *T*. *spiralis* with the plant *Arabidopsis thaliana* in Fig. 1), suggesting that this may be the ancestral mechanism of transposon silencing in animals.

At the end of this journey, the blind men’s elephant—in this instance—is revealed as a sham; the piRNAs in the model nematode *C*. *elegans* have been shown to be *faux amis*, concealing a dynamic evolutionary history whereby this broadly conserved transposon-silencing system has been lost on several occasions, in each case being supplanted by a different family of small RNAs, resourcefully produced by unrelated enzymatic mechanisms. This extraordinary recurrent wholesale turnover of an essential defensive mechanism is surprising and somewhat reminiscent of the dynamism inherent in immune systems, which fight a parallel war against selfish intruders.
